# Ossified Pulps

**Published:** 1872-09

**Authors:** S. P. Cutler


### Ossified Pulps.

BY S. P. CUTLER.

CASE 1.—I filled an upper left anterior molar for a young man 24 years old, with an ossified pulp and no sensibility. The tooth was of a dark appearance, friable and chalky; the cavity at first apparently not very extensive, but on excavating I found it very large and difficult to determine where to stop, extending down into the neck; but no pulp canal to be found. The tooth had given no trouble and was firm in the bone. I filled with amalgam and heard of no trouble afterward.

CASE 2.—A lady, 38 years old, nervous, bilious, sanguine temperament, had a filling in the mesial approximal surface of a central incisor, and a pivot tooth on its sister fang.

The filling being defective, I removed to refill. The pulp must have nearly been reached when killed, now a number of years; but now the pulp is extensively ossified, as I could easly determine with a magnifier and throwing a strong light on the tooth.

There was no sensibility only at one point toward convex, the decay having reached the enamel on the labial side, so that it readily dropped off to bottom of cavity. I built out the tooth with adhesive foil, and the tooth gave no further trouble.

CASE 3.—A young lady, 13 years old, of delicate, nervous temperament and delicate constitution, called on me in February 1870, to have her mouth attended to. On examination I found not one sound tooth in her mouth; the upper deciduous cuspids still intact and decayed slightly.

Her teeth were affected with the white chalky kind of decay, and exceedingly sensitive, rendering anything like satisfactory filling out of the question. I selected a few of the least sensitive and most favorable ones, and filled them with gold; all the others I filled with os-artificial and amalgam, after excavating in the best manner I could, thinking all the time that many of them which were very much decayed would be signal failures, intending eventually to refill all that did well. All the under front teeth were decayed and so sensitive, that I had to apply nitrate of silver before filling. Feb. 1871, I found all of her teeth so much improved by the diet, I had advised for her, such as eggs, oysters, sweet milk, and second flour, that I commenced to remove os-artificial and refill the most favorable ones. I found but little sensibility, dentine and enamel both much hardened, the os-artificial not in the least disintegrated.

By filling one or two at a setting, I had no difficulty in refilling all but the front lower teeth, which have in them the os-artificial, which I propose letting alone for a year or two longer, and then refill, or file so as to supersede the necessity of filling. Some of the back teeth with extensive decays I refilled with amalgam, the others with gold. I found all the pulps sound and apparently ossified down more or less, so much so, that the softened dentine left in at first I found clear and living. The os-artificial was a good article and almost as hard as enamel to cut away.

Case 4.—A young, healthy, married lady, with a good many fillings in her mouth, had the right superior wisdom tooth extensively decayed, and very tender with white decay. I syringed out the cavity and removed all the loose matter, and then filled with os-artificial about six weeks ago, since which time it has given no trouble. To-day, Oct. 13, '71, I removed the os-artificial which was sound, and found all undue sensibility gone; the bottom and sides of cavity while sound, firm, with evident ossification having taken place, since filling, no opening into pulp cavity as might have been predicted from the first. I refilled permanently without any capping.

Case 5.—A young lady about 19, "delicate temperament," called to have some teeth filled. The left lower anterior molar had an anterior approximal and buccal decays, the tooth rather dark in appearance, which I supposed to be dead, though it had caused no trouble. On carefuly exploring the approximal, to my surprise I found no pulp cavity as I anticipated. On the contrary, I found the "formerly nearly exposed pulp, or even quite so" ossified, firm, and hard, though dark in appearance, and slightly sensitive to cold water. The original pulp slightly projected up into the cavity bead-like.

The buccal cavity was slightly sensitive to the instrument. As all indications were favorable I filled both cavities with amalgam. Could have filled them with gold had I chosed to do so. I anticipate no trouble from this tooth. She has a few other teeth in front and back filled, though her teeth are generally good. Her father's teeth are good; her mother lost all of hers many years since; the other children have bad teeth.

Case 6.—A young lady about 17 years old, has a number of decayed teeth, several dead ones; the two superior laterals both dead, and have given trouble; the two anterior superior molars are both decayed, dead and troublesome, several others decayed. The mouth has been entirely neglected, slimy mucus covering the tooth. The second superior right molar was extensively decayed; decay reaching nerve cavity.

Instead of a cavity and dead pulp there was an ossified pulp, clear and distinct, being lighter colored than the balance of the tooth, showing distinctly the original outlines of cavity. I filled this tooth, there being slight sensibility, though had given no trouble whatever; all the dead teeth had given trouble. This case proves conclusively the advantage of ossified pulps over dead pulps in the same mouth.

Case 7.—A young lady about 15 years old has the two inferior six year molars decayed extensively, and most of crowns gone from grinding surface decays, are worn smooth from use and are worn to nerve cavities; the left one having an ossified pulp, showing clearly the shape and size of pulp cavity, the pulp presenting that peculiar pearly appearance, so often noticed in such cases, hard, firm, and not in the least sensitive. I thought best to leave it to nature.

The opposite one exactly the same kind, and extent of decay without any ossification at all, apparently the decay reaching the cavity at two points with some slight signs of sensitiveness by running down an excavator, but no uneasiness in mastication. There may be ossification going on below.

The superior six year molars I filled several years ago, but did not think the lower ones worth the trial. All the other teeth sound and healthy.

Case 8.—A gentleman over 50 years of age, has a lower right six year molar, which has two decays, posterior proximal and buccal, on the neck portion of anterior root the first large, the other small.

Both of these cavities were filled some fifteen or more years ago, but were now empty; do not know how long they have been so, though not much fresh decay.

At both points there must have been slight exposure of pulps, though no pain was ever felt. At the bottom of each cavity there was a beautiful ossification of pulp, perfectly sound, hard, and but very slightly sensitive. I refilled them with gold.

This man has most perfect teeth, none missing; he is of sound constitution, nerveous, sanguine temperament. A professional man.

In some cases we find the ossified pulp adherent to the inner walls of pulp chamber; in other cases we find the usual space surrounding nerve pulp that existed previous to the new process; this is somewhat governed by circumstances under which this process takes place. Where the teeth ossify from age or wear from friction, the first change noticed is a narrowing of canal and recession of pulp from encroachment, causing pressure and consequent absorption of pulp without the pulp itself actually hardening; in other cases the pulp alone ossifies with or without solid union to inner walls of cavity.

Case 9.—I filled a left superior second bicuspid over two years ago, on posterior proximal surface, with os-artificial over an exposed pulp, for the purpose of either procurring ossification or destruction of the pulp. At times this tooth gave some trouble, and recently had been still more troublesome.

On removing the remaining portion of filling I found the pulp projecting up into the cavity of decay, bead-like, a pearly-like nodule. There was some tenderness on pressure at first, and sensitive to cold water. I applied cotton and creosote which gave entire relief. Next day, October 17, I found the tooth entirely free from pain. I capped the bottom with spunk and creosote and filled with amalgam, without giving any uneasiness. I have hopes of this tooth as the man, a merchant, is healthy and robust, 32 years old, and of regular habits; temperament—lymphatic-bilious.

Case 10.—A young lady about 17 years of age, stout and healthy, had several front teeth filled about three years ago. The fillings in the two centrals, approximals, had been out about one year, though not much decay had taken place only in region nearest the convex, which had deepened to some extent. In the central region in both there was a slight discoloration, representing the very thin layer that must have barely protected the pulp when first filled, and which was at the time said to be sensitive. On excavacating there was now no sensibility, the dentine over the pulp solid and firm, with evident signs of a recession and ossification of cavity at those points, which fact is easily recognized by any one who has given much attention to this subject. The original decay shows the shape and site of very near exposure, at the time of first operation I apprehend no further trouble with those teeth.

The microscopic appearances are always characteristic in ossified pulps, varying somewhat though always unmistakable to a practiced observer in such cases: the irregular tubular structures; the usual plexuses and "cauda equinus" in center or near there; the irregular sizes and arrangements being always present; the original normal cavity being easily recognized under the microscope, and the one or outer region showing the original parallelism of tubes within their graceful curves and fine, short crooks. On the inner side of original land marks the irregular system referred to with combination of tubes from the one to the other system, or from the primary to the secondary series of tubulation.

Case 11.—This lady about 32 years old, had a lateral superior incisor as filled some twelve years ago; the filling having remained till about a year ago.

The cavity had no softened dentine of consequence, and not sensitive; dentine, firm and hard. At the bottom of cavity there was the former pulp exposure, perfectly ossified, showing the exact shape and extent of the same; the secondary formation hard, sound and living, and no signs of a dead pulp or tooth, which may be easily detected by a magnifyer and other means. This lady's teeth were originally soft and bad, both centrals being pivoted, others decayed and some out. Her health is good as common in this country; temperament—nervous, sanguine; rather spare built.

Case 12.—A gentleman over 60 years old, called to have a couple of teeth operated upon. He had been advised to have them treated with arsenic, although they never had caused the slightest disturbance in the way of aching. I found the two upper right bicuspids decayed, between the anterior one up to the gum, a large sized decay. On excavating I found no tenderness in either or soreness, still both presented the appearance of living teeth. The bottom of the cavity of the anterior one extended into the chamber broad cast; at the bottom of which there was presented all the appearance of an ossified canal, there being no opening or discoloration, instead the appearance of secondary dentine. At the bottom of the other cavity there stood out in bold relief the ossified pulp which had been respected by the decay, which had gracefully incircled its pearly prominence, though not as prominent as I have seen before.

I excavated and filled both, which have given no trouble since, neither are they likely to in the future. What a piece of stupid nonsense it would have been to have treated those teeth with arsenic, and that was proposed after diagnosing in the usual way.

